# A repeat unit of *Vibrio* diarrheal T3S effector subverts cytoskeletal actin homeostasis via binding to interstrand region of actin filaments

**DOI:** 10.1038/srep10870

**Published:** 2015-06-03

**Authors:** Mitsuhiro Nishimura, Takashi Fujii, Hirotaka Hiyoshi, Fumiaki Makino, Hajime Inoue, Daisuke Motooka, Toshio Kodama, Tadayasu Ohkubo, Yuji Kobayashi, Shota Nakamura, Keiichi Namba, Tetsuya Iida

**Affiliations:** 1 Research Institute for Microbial Diseases, Osaka University, 3-1 Yamadaoka, Suita, Osaka 565-0871, Japan; 2 Osaka University of Pharmaceutical Sciences, 4-20-1 Nasahara, Takatsuki, Osaka, 569-1094, Japan; 3 Graduate School of Frontier Biosciences, Osaka University, 1-3 Yamadaoka, Suita, Osaka 565-0871, Japan; 4 Riken Quantitative Biology Center, 1-3 Yamadaoka, Suita, Osaka 565-0871, Japan; 5 Graduate School of Pharmaceutical Sciences, Osaka University, 1-6 Yamadaoka Suita, Osaka 565-0871, Japan

## Abstract

A novel bacterial type III secretion effector, VopV, from the enteric pathogen *Vibrio parahaemolyticus* has been identified as a key factor in pathogenicity due to its interaction with cytoskeletal actin. One of the repeat units in the long repetitive region of VopV, named VopV_rep1_, functions as an actin-binding module. Despite its importance in pathogenesis, the manner in which the effector binds to actin and the subsequent effects on actin dynamics remain unclear. Here, we report the molecular basis of the VopV_rep1_/actin interaction. VopV_rep1_ exists as an unstructured protein in solution but potently and specifically binds filamentous actin (F-actin) and not globular actin (G-actin). The F-actin/VopV_rep1_ complex was directly visualized at 9.6-Å resolution using electron cryomicroscopy (cryoEM) and helical image reconstitution. The density map revealed the binding site of VopV_rep1_ at the interface between two actin strands, which is close to the binding site of the bicyclic heptapeptide toxin phalloidin. Consistent with this observation, VopV_rep1_ alone prevented the depolymerization of F-actin. Overall, VopV_rep1_ demonstrated unique characteristics in comparison to known actin-binding proteins, but was relatively similar to phalloidin. The phalloidin-like behavior, targeting the interstrand region of actin filaments to stabilize the filament structure, likely contributes to the pathogenicity of *V. parahaemolyticus*.

*Vibrio parahaemolyticus* is a food-borne pathogen that causes acute gastroenteritis in humans^1^. The type III secretion system (T3SS), which is the molecular machinery that delivers bacterial effectors into the cytoplasm of infected host cells, is essential for the pathogenicity of this bacterium^2^,^3^,^4^. Recent reports have shown that one of the type III secretion (T3S) effectors, VopV, plays an important role in the enterotoxicity of *V. parahaemolyticus*^5^,^6^. Among the three repeat units within the long repeat region in VopV, VopV_rep1_ has been identified as the key unit for F-actin binding and VopV enterotoxicity (Fig. 1(a); UniProt: Q87GF9)^5^,^7^.

The actin cytoskeleton is involved in various cellular processes, and its behavior is strictly controlled by numerous actin-binding proteins and their regulators^8^,^9^,^10^.To date, a lot of actin-binding proteins have been identified; these proteins affect cellular actin dynamics in various ways, such as sequestering actin monomers, severing or capping the filaments, forming networks or bundles of filaments, and supporting nucleation of filament formation^10^. These different interactions are mediated by individual actin-binding motifs and correspond to the functional diversity of the actin-protein interactions^11^,^12^. A number of pathogenic bacteria abuse the versatile ability of actin cytoskeleton to promote its infection^13^,^14^. Effectors take control of actin cytoskeleton by directly interacting with the actin molecule as actin-binding proteins or by indirectly interfering with the actin architecture via its regulation system^14^,^15^.

Despite our increasing knowledge of actin-binding proteins including bacterial effectors, VopV_rep1_ has no sequence homology with known actin-binding proteins^5^ and thus, researchers are not sure how VopV_rep1_ interacts with actin or how VopV_rep1_ affects actin dynamics. Here, we analyzed the molecular characteristics of VopV_rep1_ as a novel actin-binding protein. Direct visualization of the interaction between F-actin and VopV_rep1_ using electron cryomicroscopy (cryoEM) revealed the unique binding mechanism of VopV_rep1_ and demonstrated that VopV_rep1_ had a stabilizing effect on F-actin.

## Results

### VopV_rep1_ exists as an unstructured protein in solution, but potently and specifically binds F-actin

**Figure 1:**
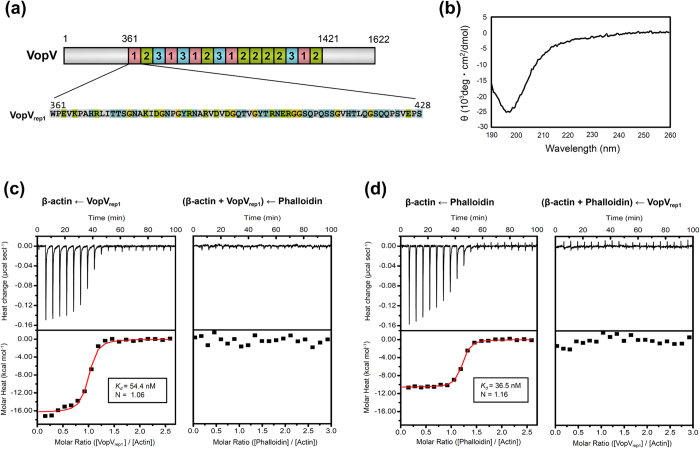
VopV_rep1_ was an unstructured protein that demonstrated high affinity for F-actin. (**a**) The repeating structure of VopV was composed of three patterns of repeat sequences. The VopV_rep1_ sequence used in this study is shown below, and the residues are color-coded by their hydrophobicity (charged: green; polar: cyan; glycine: yellow; and hydrophobic: gray) to emphasize the high proportion of hydrophilic residues. (**b**) Circular dichroism analysis of VopV_rep1_ revealed a typical random coil spectrum with a minimum peak at approximately 196 nm. (**c**), (**d**) The binding affinities of VopV_rep1_ and phalloidin with cytoskeletal actin were analyzed using isothermal titration calorimetry. The dissociation constant (*K*_*d*_) and binding stoichiometry (N) obtained by curve fitting analysis are shown in the inset. The binding pattern of VopV_rep1_ (c, left) was similar to that of phalloidin (d, left). After the addition of VopV_rep1_, phalloidin did not bind to actin (c, right), as was observed for the addition of VopV_rep1_ to phalloidin-saturated actin (d, right).

First, we overexpressed a construct containing VopV_rep1_ (residues 361–428) and purified the resulting recombinant protein. The circular dichroism spectrum of VopV_rep1_ was clearly indicative of a random coil pattern (Fig. 1(b)), demonstrating that the recombinant VopV_rep1_ did not form a stable fold in solution. Indeed, the high proportion of hydrophilic, polar, and glycine residues in VopV_rep1_ (~67.6%, Fig. 1(a)) was consistent with the typical characteristics reported for intrinsically unstructured proteins^16^. To assess whether the recombinant protein bound to actin, the binding affinity of VopV_rep1_ to cytoskeletal actin was determined using isothermal titration calorimetry (ITC). Without a stable fold in solution, VopV_rep1_ bound to actin with high affinity (*K*_*d*_ = 54.4 nM) at a binding stoichiometry of N = 1.06, which is comparable to the well-known F-actin-binding toxin phalloidin^17^,^18^, which has a binding affinity of *K*_*d*_ = 36.5 nM and a binding stoichiometry of N = 1.16 in our experiment (Fig. 1(c,d) and Supplementary Table S1). These values indicate that their stoichiometries are both 1.0. The affinity of VopV_rep1_ was as high as or higher than that of other well-studied actin-binding proteins/peptides, such as profilin (*K*_*d*_ = 0.1–5 μM)^19^, ADF/cofilin (*K*_*d*_ = 0.1–10 μM)^19^, Wiskott-Aldrich syndrome protein homology domain 2 peptides (*K*_*d*_ = 0.052–27 μM)^20^, and the nebulin repeat fragment (*K*_*d*_ = 220 μM)^21^, suggesting that VopV_rep1_ was well adapted for actin binding. Because actin exists in a dynamic equilibrium between F-actin and G-actin, we next examined the binding preference of VopV_rep1_ to each form of actin. An increase in the G-actin/F-actin ratio induced by the F-actin depolymerizing toxin latrunculin^22^ caused a decrease in the binding stoichiometry of VopV_rep1_ to N = 0.28 (Supplementary Fig. S1 and Table S1), indicating that VopV_rep1_ preferentially bound F-actin. Conversely, an increase in the F-actin/G-actin ratio using the F-actin-stabilizing toxin phalloidin^18^,^23^,^24^ unexpectedly resulted in complete inhibition of the actin/VopV_rep1_ interaction (Fig. 1(c)). One possible explanation for these observations is that the bindings of phalloidin and VopV_rep1_ are mutualy exclusive. The observation that VopV_rep1_ conversely prevented the binding of phalloidin to actin (Fig. 1(d)) supported this possibility.

### Direct visualization of the VopV_rep1_/F-actin interaction by cryoEM

**Figure 2:**
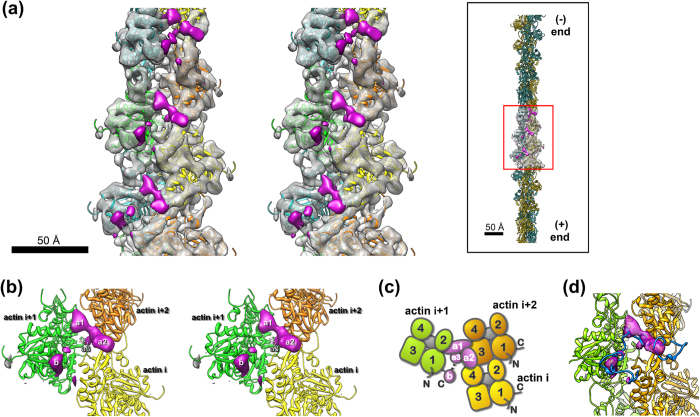
Structure of the F-actin/VopV_rep1_ complex visualized by cryoEM image analysis at 9.6-Å resolution. (**a**) A magnified view of the F-actin/VopV_rep1_ complex in stereo. The long axis of the actin filament is vertical, as shown in the inset. The density map is represented as a transparent envelope and is filled by actin models that are alternately colored cyan/green and orange/yellow according to the two actin strands. The extra regions that likely correspond to VopV_rep1_ are colored magenta. For clarity, the extra density derived from the N-terminal region of actin was colored dark gray. Three repetitive clusters are observed in this view. (**b**) Detailed stereo view of the VopV_rep1_ densities on the F-actin model. Three distinguishable densities, a1, a2, and b, were in close contact with three actin units i, i + 1, and i + 2. (**c**) A schematic representation of the relative spatial arrangement of the VopV_rep1_ densities (magenta) and surrounding actin domains numbered 1-4 with ATP/ADP (gray). (d) A binding model of VopV_rep1_ (blue) in an elongated form, fitted to the observed densities. The chains outside the densities would be unstructured.

We then used cryoEM image analysis to elucidate the mode of VopV_rep1_ binding to cytoskeletal F-actin at the molecular level; the unstructured nature of VopV_rep1_ and the filamentous structure of actin made it nearly impossible to use other approaches, such as single-crystal X-ray crystallography or nuclear magnetic resonance (NMR) spectroscopy. Recent advances in the cryoEM method applied to F-actin^25^ have enabled the direct visualization of the human cytoskeletal actin/VopV_rep1_ complex at 9.6-Å resolution (Fig. 2(a)). The densities corresponding to the actin molecules were readily assigned to the atomic model of skeletal muscle F-actin without significant conformational change from the previously determined structure (Fig. 2(a) and Supplementary Fig. S2). One apparent exception was observed at the N-terminal region, which oriented in different direction from the model (Supplementary Fig. S2). One possible cause is the difference in sequence between cytoskeletal actin (β- and γ-actins, in this study) and skeletal muscle actin (α-actin, Fujii, *et al.* 2010)^25^. We should also note that the N-terminal structure of actin is also not observed in recent high resolution studies of F-actin composed of α-actin from striated muscle^26^,^27^. In addition to the density of the actin filament, densities corresponding to VopV_rep1_ were clearly observed along the interface of the two parallel actin strands in a repetitive manner (Fig. 2(a,b), colored magenta). One major density (sites a1-a2) was located at the cleft between the two actin strands (Fig. 2(a,b)), and an additional density (site b) was found immediately adjacent to the C-terminal region of one actin molecule (actin i + 1; Fig. 2(b,c)). Therefore, the VopV_rep1_ densities simultaneously contacted three actin molecules. The major elongated density of sites a1-a2 of VopV_rep1_ occupied the interface surrounded by the three actin subunits. There is a density extended from the connecting region between densities a1-a2 into the filament interior (named a3 in Fig. 2(b,c)), which is located deep inside the interstrand cleft. At the a1 site, the density was contacted domains 1 and 2 of an actin i + 1 molecule (Fig. 2(b,c)). The density extended toward the opposite actin strand, reaching domain 3 of actin i + 2 and further stretched to domain 4 of another actin i molecule running across the longitudinal interface (Fig. 2(b,c)). The additional density at site b was located near the C-terminal region at domain 1 of actin i + 1 around the extension of the interface of two actin strands (Fig. 2(b,c)). Because of the limited resolution of the map, we used the atomic model of actin (PDB ID: 3MFP)^25^ to predict the actin residues involved in the interaction with VopV_rep1_ (Supplementary Fig. S3 and Table S2). The density a3, which is located in the interior of actin filament, is close to the binding site of phalloidin^18^,^24^ (indicated by asterisk in Fig. 2(b)). Because the binding stoichiometry was determined by ITC analysis, the densities described above (i.e., sites a1, a2, a3, and b) are likely to correspond to one VopV_rep1_ molecule. The size of VopV_rep1_, 68 residues, was more than sufficient to explain the observed densities, suggesting that some regions connecting the densities were still unstructured and therefore not visible in the map. At a lower threshold, weak densities extending and connecting the major densities are observed (Supplementary Fig. S4). The volume ratio of the extra densities to the actin density is 7.7% and 13.0% at the high and low threshold, respectively. As a coarse estimation, the mass for VopV_rep1_ includes 29 to 48 residues (calculated from the residue number of actin), meaning that part of 68 residues of VopV_rep1_ is invisible in this reconstitution due to disorder. This concept was consistent with its natively unstructured properties, as demonstrated in a simulated model (Fig. 2(d)).

### Bound VopV_rep1_ stabilizes the F-actin protecting from depolymerization.

**Figure 3:**
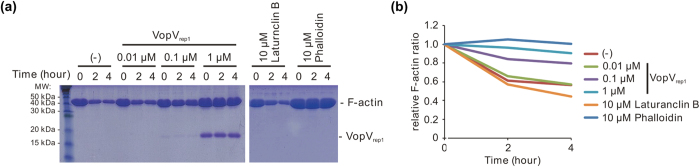
F-actin protection from depolymerization by VopV_rep1_. (**a**) F-actin stabilization was examined using a high-speed cosedimentation assay. A mixture of depolymerizing F-actin in the absence or presence of VopV_rep1_, latrunculin B, and phalloidin was ultracentrifuged after 0, 2, or 4 h incubation. The pellet fractions (F-actin) were separated using SDS-PAGE, and the gels were stained with Coomassie blue. (**b**) The stabilized F-actin shown in (**a**) was analyzed using densitometry. The relative F-actin ratio was calculated as the amount of F-actin pelleted at each time point divided by that of zero time point amount.

In the density map, VopV_rep1_ occupied a key position surrounded by three actin monomers that reinforced both the lateral and longitudinal interactions within the filament, suggesting the ability of VopV_rep1_ to stabilize F-actin. In addition to the observed binding mode, the specific, high-affinity recognition of F-actin results in F-actin stabilization^19^. As expected, the F-actin-stabilizing activity of VopV_rep1_ was demonstrated by the observation that VopV_rep1_ strongly protected the actin filament from depolymerization in low-salt conditions (Fig. 3) to the same extent as phalloidin. These results suggested that VopV_rep1_ played a role in anchoring cytoskeletal actin and simultaneously interfered with actin dynamics by stabilizing F-actin.

## Discussion

In this study, a series of biophysical analyses of VopV_rep1_ revealed its peculiar characteristics as an actin-binding protein. VopV_rep1_ bound across two actin strands; therefore, the binding likely depended on the higher-order structure of F-actin. Such a binding mode may account for the specificity of F-actin observed in the ITC analysis. In particular, the binding site of VopV_rep1_ did not include the hydrophobic cleft between domains 1 and 3 of the actin molecule (Fig. 2(c)), which is recognized by the vast majority of actin-binding proteins^12^. The interstrand region of the actin filament is known to be used as a binding site of the muscle actin-stabilizing factor nebulin^28^ and the *Salmonella*-derived T3S effector SipA^29^. The actin-binding domain of SipA (SipA_446–684_, 238 residues) is significantly larger than VopV_rep1_, and the domain itself possesses a stable fold^30^, in contrast to the intrinsically unstructured VopV_rep1_. Electron microscope image analysis of the F-actin/SipA_446-684_ complex has revealed that the binding site of SipA_446-684_ involves a more extended region in addition to the filament interface^29^. Conversely, although the actin-binding fragment of nebulin is relatively small (~35 residues) and intrinsically unstructured in solution^21^, as is VopV_rep1_, the affinity of the nebulin fragment to F-actin is significantly weaker than that of VopV_rep1_ (nebulin, *K*_*d*_ = 200–500 μM vs. VopV_rep1_, *K*_*d*_ = 54.4 nM)^21^. The actin-binding motif of nebulin (SDxxYK)^21^ was not present in the VopV_rep1_ sequence. Therefore, our data demonstrated that VopV_rep1_ possessed novel characteristics as an actin-binding protein/peptide, targeting the interstrand interface of F-actin with significantly high affinity through its intrinsic unstructured nature.

F-actin stabilization by VopV_rep1_ appears to be at least partially responsible for the observed anomaly of cytoskeletal actin in *V. parahaemolyticus*-infected small intestinal epithelial cells^31^, supported by the observation that the actin cytoskeleton in microvilli, which are the target of *V. parahaemolyticus* infection, is in dynamic equilibrium^32^ and is thus susceptible to perturbation by extrinsic factors. The homeostasis of F- and G-actin is a well-known target for pathogenic effectors^15^. With regard to *V. parahaemolyticus*, VopL is known to possess F-actin nucleation activity^33^,^34^; thus, VopV and VopL likely facilitate the excessive formation of F-actin in a concerted manner.

VopV_rep1_ is a small, flexible unit that is a stand-alone module properly tailored for binding and stabilizing F-actin. Full-length VopV contains five homologous VopV_rep1_ units separated by two other repetitive units (Fig. 1(a))^5^, which simultaneously but individually act on F-actin, resulting in even higher affinity or leading to actin bundling, as observed in VopV-affected cells^5^. Its behavior as an actin-binding unit and the phalloidin-like potency of VopV_rep1_ suggest that this sequence may have versatile applications in cell biology studies, particular when considering the broad use of phalloidin as an imaging tool to monitor F-actin. In combination with genetic engineering techniques, VopV_rep1_ could be employed as an inducible factor to target F-actin and cause F-actin stabilization or actin bundling (using a tandem arrangement with an appropriate spacer). Such applications would be useful for the analysis of actin dynamics, F-actin structure^35^, and programmed cell death via F-actin abnormalities in living cells in tissues^36^.

Advanced techniques in cryoEM image analysis revealed the interaction of VopV_rep1_ with F-actin, which is the primary molecular step in the pathogenicity of *V. parahaemolyticus*. Unexpectedly, VopV was found to target a similar site as phalloidin and demonstrated a unique binding mode for F-actin that differed from that of known actin-binding proteins. The unstructured nature of VopV_rep1_ may contribute to its ability to enter the elongated binding site at the interstrand region of F-actin while maintaining its affinity and specificity. This novel and well-organized binding mode for VopV_rep1_ provides a glimpse into the unusual association between the pathogenic effector of *V. parahaemolyticus* and the fundamental human cytoskeleton.

## Methods

For details of the MD calculation for making simulated model of VopV_rep1_ see Supplemental Methods.

### Overexpression and purification of VopV_rep1_

The VopV_rep1_ sequence was subcloned into a pET-32b vector (Novagen) containing a modified multiple cloning site to express a fusion protein composed of thioredoxin, His_6_, and a thrombin protease site followed by VopV_rep1_. BL21(DE3) cells (Novagen) were transformed with this vector and cultivated in LB medium supplemented with 100 mg/mL ampicillin at 37 °C. Protein expression was induced with 0.2 mM IPTG, and cells were further incubated at 20 °C for 6 h. The cells were harvested by centrifugation, flash frozen in liquid N_2_, and thawed on ice. The cells were then resuspended in lysis buffer (20 mM Tris-HCl, pH 7.5, and 500 mM NaCl), treated with 0.2% Triton X-100 for 20 min on ice, and disrupted by ultrasonication. The cell debris was then removed by centrifugation. The supernatant was applied to a Ni-charged HiTrap Chelating column (GE Healthcare) equilibrated with lysis buffer. The target protein was eluted with lysis buffer containing 300 mM imidazole. The fusion tags were removed through thrombin cleavage (Sigma-Aldrich) for 18 h at 25 °C during dialysis in Tris-HCl (pH 7.5) and 5 mM NaCl. The VopV_rep1_ protein was further applied to a HiTrap SP column (GE Healthcare) and eluted using a 5–300 mM NaCl gradient. After concentration using an Amicon ultra concentrator (Millipore), VopV_rep1_ was further purified by size-exclusion chromatography using a HiLoad 26/60 Superdex 75 prep grade column (GE Healthcare). The purity was verified using SDS-PAGE analysis. Purified VopV_rep1_ was stored at –80 °C prior to use.

### Circular dichroism (CD) spectroscopy

VopV_rep1_ was dialyzed in 20 mM Tris-HCl (pH 7.2). The CD spectrum of 125 μM VopV_rep1_ was acquired using a JASCO J-720 spectropolarimeter in a 1-mm quartz cell at 20 °C.

### Isothermal titration calorimetry (ITC)

VopV_rep1_ and cytoskeletal actin were dialyzed in 30 mM Tris-HCl (pH 7.5) and 75 mM KCl. The titration experiment was conducted using a MicroCal iTC200 calorimeter. The measurement was performed in general actin buffer (20 mM Tris-HCl, pH 7.5, 50 mM KCl, 1 mM MgCl_2_, and 0.2 mM ATP) at 25 °C. It was assumed that almost all the actin molecules formed F-actin in this solution. Phalloidin was dissolved in DMSO and used at a concentration of 110 μM in general actin buffer containing 1% DMSO. The sample cell was filled with 8.8 μM actin, and 110 μM VopV_rep1_ or phalloidin was loaded in the titration syringe. To account for the diluted DMSO in the case of its use as a drug, 1% DMSO was added to the actin solution. An inhibition assay was performed using a sequential titration experiment in which the actin solution that was titrated by the first binding sample, that is, VopV_rep1_ or phalloidin, was used for the second titration assay with phalloidin or VopV_rep1_, respectively. The obtained data were analyzed using Origin 7 Software (OriginLab Corporation) and fitted using a one-site binding model.

### CryoEM

#### Image collection

The F-actin/VopV_rep1_ sample retrieved after ITC titration (12.5 μM VopV_rep1_, 7.8 μM actin) was used for cryoEM image data collection and analysis. The experimental procedures for cryoEM were identical to those of a previously published report^25^. Briefly, the sample solution was loaded on Quantifoil holey carbon molybdenum grid (R0.6/1.0, Quantifoil) and plunge-frozen into liquid ethane using Vitrobot (FEI). Image collection was performed at temperature of 50–60 K using a JEOL JEM3200FSC electron microscope equipped with a liquid helium-cooled specimen stage, an Ω-type energy filter, and a field-emission electron gun operated at 200 kV. In total, 445 images were recorded on a CCD camera (TemCam-F415MP, TVIPS) at magnification of around 170,100× and a defocus range of 1.0–2.4 μm. The image pixel size corresponded to 1.677 Å.

#### Image analysis

Image analysis using the iterative helical real-space reconstitution method (IHRSR)^37^ was performed using EMAN 1.93^38^ and SPIDER 15.06^39^. The defocus and astigmatism of each image were determined using CTFFIND^40^. In total, 39,910 image segments of F-actin were boxed, and ~121,000 actin molecules were included. Images were corrected for phase and amplitude contrast transfer function (CTF). The images were aligned and cross-correlated to a series of reference projection images, and a 3D image was reconstituted by back projection. The 3D images were improved by imposing helical symmetry parameters in an iterative manner and refined until convergence.

#### Model analysis

The rabbit α-skeletal actin structure (PDB ID: 3MFP) was fitted to the density, and symmetrical actin units were generated using UCSF Chimera software^41^. In the iterative process of image analysis, the helical symmetry and axial repeat distance were refined and converged to a subunit rotation of –166.8° and an axial repeat of 27.6 Å, which resulted in a helical symmetry of approximately 41 subunits/19 turns (~2.158). This value of the F-actin/VopV_rep1_ complex corresponded to a helical structure slightly overtwisted from that of native F-actin, which was reported in a study of rabbit skeletal muscle actin with approximately 67 subunits/31 turns (~2.161)^25^ and was relatively close to the phalloidin-bound form of F-actin with approximately 69 subunits/32 turns (~2.156)^18^.

### F-actin stabilization assay

F-actin stabilization was performed using the Actin Binding Protein Biochem Kit (Cytoskeleton Inc.). Briefly, F-actin was pre-assembled from purified human platelet nonmuscle actin (1 mg/mL) at room temperature for 1 h in F-buffer containing 0.2 mM CaCl_2_, 50 mM KCl, 2 mM MgCl_2_, and 1 mM ATP in 5 mM Tris-HCl (pH 8.0). The assembled F-actin was then depolymerized by a 1:5 dilution with G-buffer (0.2 mM CaCl_2_ in 5 mM Tris-HCl, pH 8.0) in the absence or presence of VopV_rep1_, latrunculin, and phalloidin under the indicated final concentrations. After 0, 2, or 4 h of incubation on ice, the samples were ultracentrifuged (120,000 × *g* for 2 h) at 4 °C. The pellets were analyzed using SDS-PAGE, and the gels were stained with Coomassie blue. The density of the pelleted F-actin in individual fractions was determined using ImageJ software (National Institutes of Health).

## Additional Information

**How to cite this article**: Nishimura, M. *et al.* A repeat unit of *Vibrio* diarrheal T3S effector subverts cytoskeletal actin homeostasis via binding to interstrand region of actin filaments. *Sci. Rep.* **5**, 10870; doi: 10.1038/srep10870 (2015).

## Supplementary Material

Supplementary Information
