# Supplementary material for: A repeat unit of Vibrio diarrheal T3S effector subverts cytoskeletal actin homeostasis via binding to interstrand region of actin filaments
Source: Sci Rep. 2015 Jun 3;5:10870. doi: 10.1038/srep10870 (PMC4650670; doi:10.1038/srep10870)
Supplement: Supplementary Information [file srep10870-s1.doc]

Title

A repeat unit of *Vibrio* diarrheal T3S effector subverts cytoskeletal actin homeostasis via binding to interstrand region of actin filaments.

Author Affiliations

Mitsuhiro Nishimura,^a,b^, Takashi Fujii,^c^ Hirotaka Hiyoshi,^a^, Fumiaki Makino,^c^, Hajime Inoue,^b^ Daisuke Motooka,^a,d^ Toshio Kodama,^a^ Tadayasu Ohkubo,^d^ Yuji Kobayashi,^b^ Shota Nakamura,^a^ Keiichi Namba,^c,e^, and Tetsuya Iida,^a*^

^a^Research Institute for Microbial Diseases, Osaka University, 3-1 Yamadaoka, Suita, Osaka 565-0871, Japan

^b^Osaka University of Pharmaceutical Sciences, 4-20-1 Nasahara, Takatsuki, Osaka, 569-1094, Japan

^c^Graduate School of Frontier Biosciences, Osaka University, 1-3 Yamadaoka, Suita, Osaka 565-0871, Japan

^d^Graduate School of Pharmaceutical Sciences, Osaka University, 1-6 Yamadaoka Suita, Osaka 565-0871, Japan

^e^Riken Quantitative Biology Center, 1-3 Yamadaoka, Suita, Osaka 565-0871, Japan

^*^Correspondence: iida@biken.osaka-u.ac.jp

Supplementary Figures


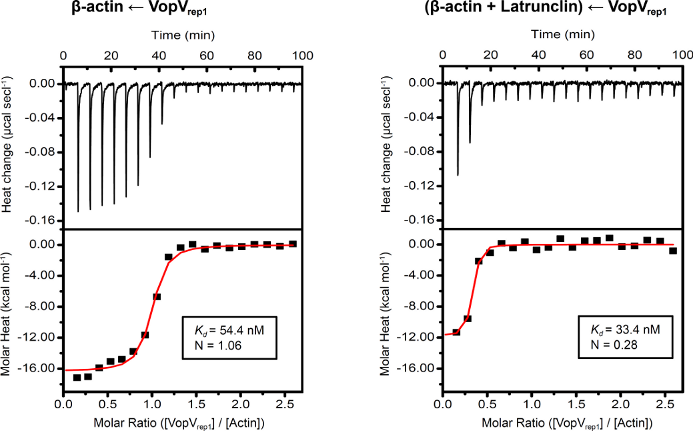


Figure S1. **F-actin depolymerization by latrunculin caused a decrease in the actin population to which VopV_rep1_ bound.** The interaction of VopV_rep1_ with latrunculin-treated actin was analyzed using isothermal titration calorimetry (right). As a reference, the binding profile for untreated actin is also shown (left). After the addition of latrunculin, the binding stoichiometry N was dramatically decreased to 0.28 (right), in contrast to the binding stoichiometry of N = 1.06 for untreated actin (left), indicating an increase in the inability of VopV_rep1_ to bind G-actin as a result of the F-actin depolymerization caused by latrunculin.


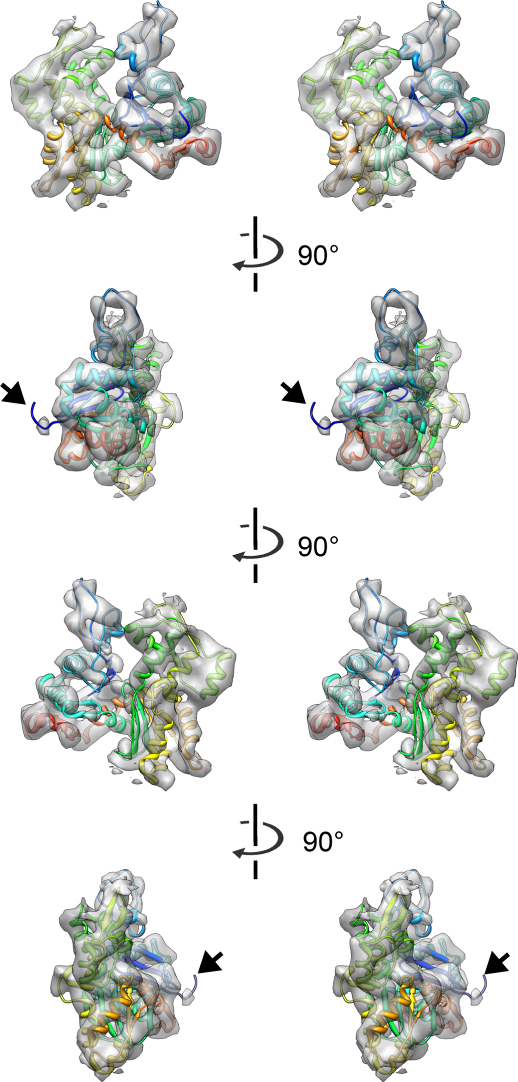


Figure S2. **The goodness of fit between the actin model and the density observed for the F-actin/VopV_rep1_ complex.** The previously determined structure of rabbit skeletal actin in F-actin (PDB ID: 3MFP) was fitted to the density of the F-actin/VopV_rep1_ complex. Because the F-actin used in this study was predominantly the β isoform, the mismatched residues between α and β isoforms were substituted. As shown, the actin model fits the density well without any conformational rearrangement regardless of the isoform difference, except for the N-terminal region indicated by arrow.


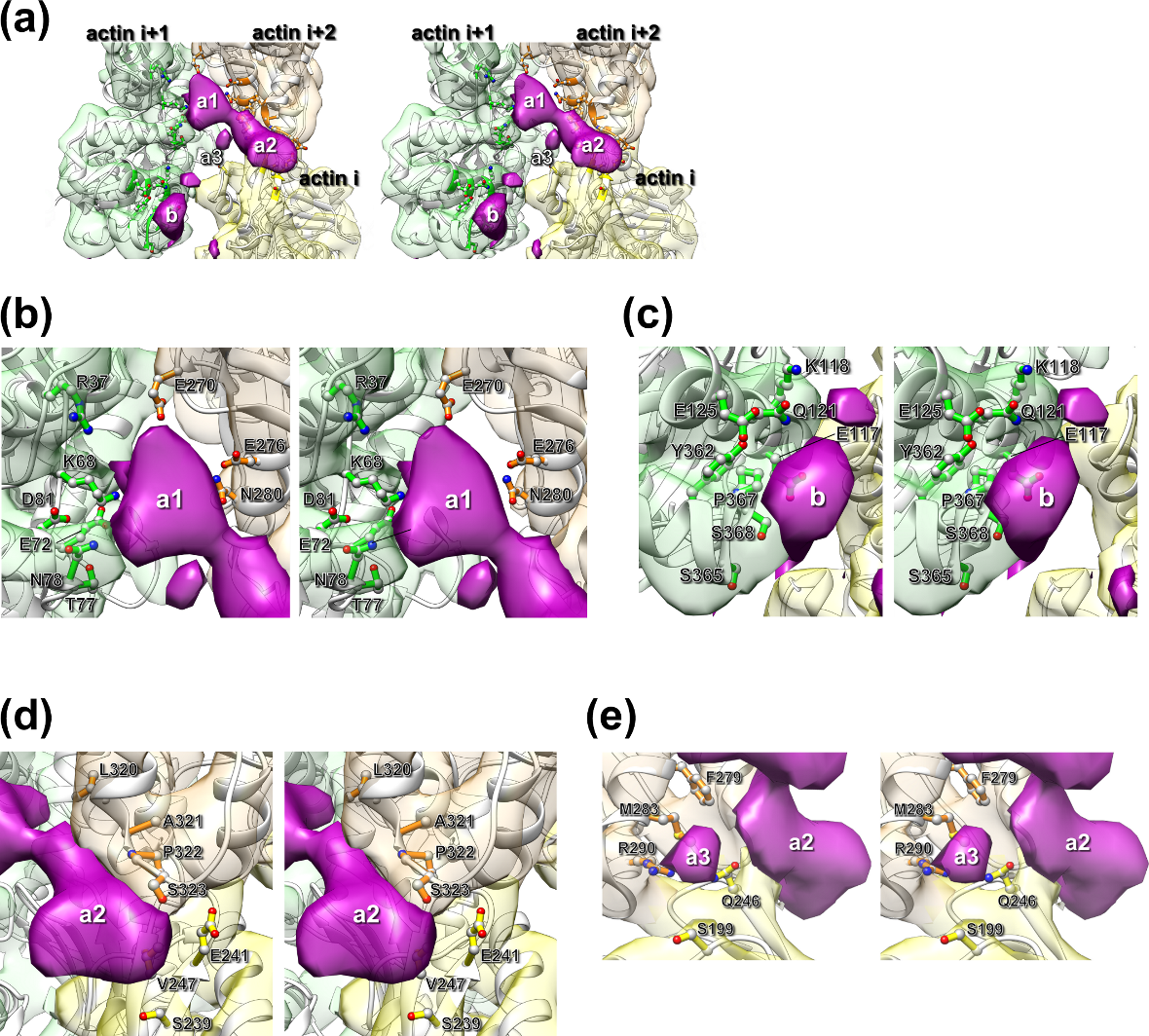


Figure S3. **Detailed views of the interaction site between F-actin and VopV_rep1_.** Overall view (a) and the focused views for the sites around density a1 (b), density b (c), density a2 (d) and density a2 and a3 (e) are shown. Note that (e) is viewed from the inner side of the actin filament. Actin residues that are close to the VopV_rep1_ densities and therefore expected to be involved in the interaction with VopV_rep1_ are depicted as a ball-and-stick model. All views are stereo images.


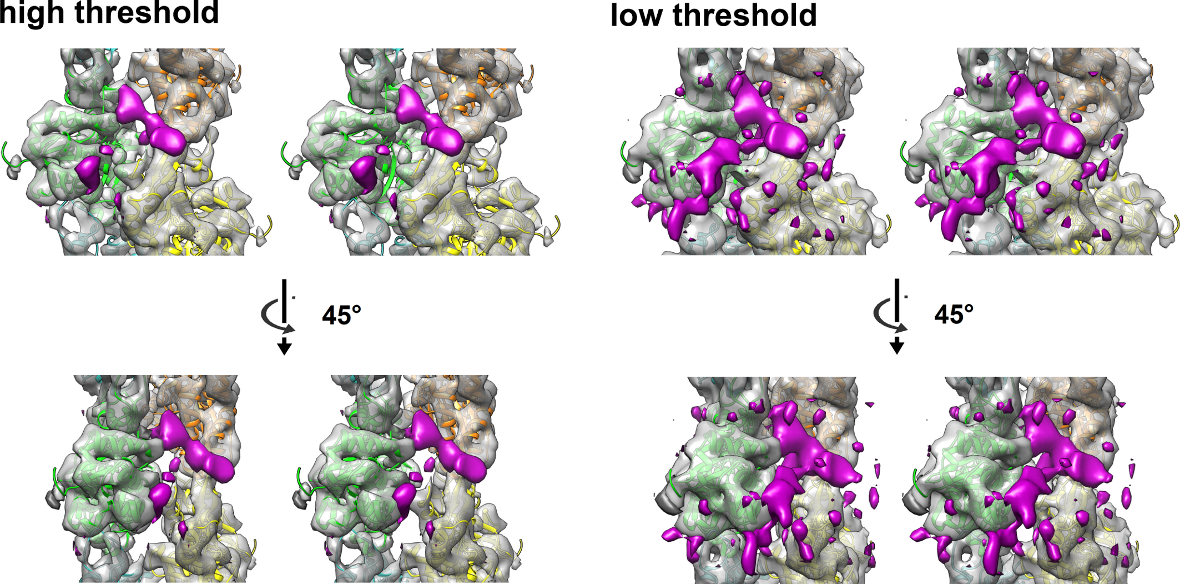


Figure S4. **Weak densities of VopV_rep1_ were observed at low threshold level.**

Densities corresponding to actin (gray) and extra densities (magenta) were shown at different threshold levels. At the low threshold (right), the weak densities occupying the filament interface are visible. All of the views are stereo images.

Supplementary Tables

**Table S1. Thermodynamic parameters of the interaction between cytoskeletal actin and VopV_rep1_**.

|  | N | *K_a_*, μM (*K_d_*, nM^-1^) | Δ*H*, kcal mol^-1^ | ^*^*T*Δ*S*, kcal mol^-1^ |
| --- | --- | --- | --- | --- |
| Actin |  |  |  |  |
| ^†^VopV_rep1_ | 1.06. ± 0.12 | 15.9 ± 4.1 (62.9) | -16.3 ± 0.30 | -6.39 |
| ^‡^Phalloidin | 1.16 ± 0.004 | 27.4 ± 3.1 (36.5) | -10.6 ± 0.07 | -0.41 |
| Actin-Latrunculin |  |  |  |  |
| ^‡^VopV_rep1_ | 0.28 ± 0.012 | 29.9 ± 1.8 (33.4) | -11.8 ± 0.70 | -0.16 |
| Actin-VopV_rep1_ |  |  |  |  |
| Phalloidin | NB | NB | NB | NB |
| Actin-Phalloidin |  |  |  |  |
| VopV_rep1_ | NB. | NB | NB | NB |

NB represents “no binding” due to the absence of a thermal response.

^*^The change in entropy (*T*Δ*S*) was calculated from *K_a_* and Δ*H* using the equations Δ*G* = -RT ln (1/*K_a_*) and Δ*G* = Δ*H* – *T*Δ*S*.

^†^Standard deviation for three experiments was shown for each parameter.

^‡^Fitting errors were shown for each parameter.

**Table S2. Actin residues close to the VopV_rep1_** densities.

| VopV_rep1_ density† | Actin residue | Actin domain | Actin position† |
| --- | --- | --- | --- |
| a1 | R37 | 2 | i+1 |
|  | K68 | 2 | i+1 |
|  | E72 | 1 | i+1 |
|  | T77 | 1 | i+1 |
|  | N78 | 1 | i+1 |
|  | D81 | 1 | i+1 |
|  | E270 | 3 | i+2 |
|  | E276 | 3 | i+2 |
|  | N280 | 3 | i+2 |
| a2 | F279 | 3 | i+2 |
|  | M283 | 3 | i+2 |
|  | L320 | 3 | i+2 |
|  | A321 | 3 | i+2 |
|  | P322 | 3 | i+2 |
|  | S323 | 3 | i+2 |
|  | S199 | 4 | i |
|  | S239 | 4 | i |
|  | E241 | 4 | i |
|  | Q246 | 4 | i |
|  | V247 | 4 | i |
| a3 | M283 | 3 | i+2 |
|  | R290 | 3 | i+2 |
|  | Q246 | 4 | i |
| b | E117 | 1 | i+1 |
|  | K118 | 1 | i+1 |
|  | Q121 | 1 | i+1 |
|  | E125 | 1 | i+1 |
|  | Y362 | 1 | i+1 |
|  | S365 | 1 | i+1 |
|  | P367 | 1 | i+1 |
|  | S368 | 1 | i+1 |

^†^ Definitions are given in the legends for Fig. 2(c).

Supplemental Methods

**MD calculation of VopV_rep1_ conformational ensembles and model construction**

To investigate the possible chain conformations of VopV_rep1_, we performed a two-step MD simulation using the Gromacs 4.5.4 package^42^ with the amber03 force field^43^. The initial linear strand structure of VopV_rep1_ was constructed by connecting amino acid residues using the Tinker package^44^. The initial structure was solvated in a rectangular box of water molecules. An additional 113 K^+^ and 116 Cl^-^ ions were added to the solvent as counter ions to neutralize the entire system. The final system size was approximately 150 Å × 25 Å × 250 Å, which was composed of 184,877 atoms and 61,212 TIP3P^45^ water molecules. After 1,000 steps of energy minimization, a 1-ns MD simulation was performed with the NTV ensemble at T = 300 K. After the first MD simulation, the final structure was placed in a hexahedron box (50 Å × 50 Å × 50 Å) with 2,996 TIP3P water molecules, 8 K^+^ ions, and 11 Cl^-^ ions. After 1,000 steps of energy minimization, 20-ps simulated annealing (SA) was performed using the following protocol. The system temperature was heated to 800 K for 5 ps, maintained at 800 K for 10 ps, and subsequently cooled to 300 K for 5 ps. After SA, a 10-ns MD simulation (time step of 2.0 fs) was performed with the NTV ensemble at T = 300 K. Periodic boundary conditions were employed on the hexahedron simulation box. A constant temperature was set using a V-rescale thermostat with a time constant of 0.1 ps. The long-range electrostatic interactions, which were calculated using the particle-mesh Ewald method^46, 47^ and the Lennard-Jones interactions, were cut off at 12 Å. All hydrogen bond lengths were constrained to their equilibrium length using the LINCS algorithm^48^. During the course of the 10 ns MD trajectory, 100 conformations were obtained every 100 ps. Model fitting of the conformational ensemble in the electron density map was performed using the UCSF Chimera software^41^. Among the models with the highest overlapping score, one of the most plausible models without large clashes with the actin molecule was selected as an assumed binding model in an arbitrary manner.

Supplemental References

1. Hess, B., Kutzner, C., van der Spoel, D. & Lindahl, E. GROMACS 4: Algorithms for highly efficient, load-balanced, and scalable molecular simulation. *J. Chem. Theory Comput.* **4**, 435-447 (2008).
2. Duan, Y., Wu, C., Chowdhury, S., Lee, M.C., Xiong, G. *et al.* A point-charge force field for molecular mechanics simulations of proteins based on condensed-phase quantum mechanical calculations. *J. Comput. Chem.* **24**, 1999-2012 (2003).
3. Ren, P., Wu, C. & Ponder, J.W. Polarizable Atomic Multipole-based Molecular Mechanics for Organic Molecules. *J. Chem. Theory Comput.* **7**, 3143-3161. (2011).
4. Jorgensen, W.L., Chandrasekhar, J., Madura, J.D., Impey, R.W. & Klein, M.L. Comparison of simple poteintial functions for simulating liquid water. *J. Chem. Phys.* **79**, 926-935 (1983).
5. Darden, T., York, D. & Pedersen, L. Particle mesh Ewald: An NLog(N) method for Ewald sums in large systems. *J. Chem. Phys.* **98**, 10089-10092 (1993).
6. Essmann, U. *et al.* A smooth particle mesh Ewald method. *J. Chem. Phys.* **103**, 8577-8593 (1995).
7. Hess, B., Bekker, H., Berendsen, H.J.C. & Fraaije, J.G.E.M. LINCS: A linear constraint solver for molecular simulations. *J. Comput. Chem.* **18**, 1463-1472 (1997).
